# Explaining geographic patterns of suicide in the US: the role of firearms and antidepressants

**DOI:** 10.1186/2197-1714-1-6

**Published:** 2014-03-20

**Authors:** April Opoliner, Deborah Azrael, Catherine Barber, Garrett Fitzmaurice, Matthew Miller

**Affiliations:** 1 Department of Epidemiology, Harvard School of Public Health, 677 Huntington Ave, Boston, MA 02115 USA; 2 Department of Health Policy and Management and Harvard Injury Control Research Center, Harvard School of Public Health, 677 Huntington Ave, Boston, MA 02115 USA; 3 Department of Biostatistics, Harvard School of Public Health, McLean Hospital, 677 Huntington Ave, Boston, MA 02115 USA

## Abstract

**Background:**

Suicide rates vary more than 3-fold across the fifty states. Previous ecological studies have pointed, separately, to covariation of suicide mortality with rates of a) household firearm ownership, and b) antidepressant prescriptions.

**Methods:**

An ecologic study using panel data from 2001-2005 was used to evaluate the joint and separate association of household firearm ownership and antidepressant prescription rates with the distribution of suicide rates across the United States. Key exposures were household firearm ownership prevalence (using data from the 2004 Behavioral Risk Factor Surveillance System) and antidepressant prescription rates (using data supplied by IMS health). Negative binomial mixed-effect models were used to estimate the association between household firearm ownership prevalence and antidepressant prescriptions rates and state level suicide rates (using data from the National Vital Statistics System), overall and by method of suicide (firearm vs. non-firearm). Sensitivity analyses examined analogous county-level data for those counties for which firearm ownership measures were available. All analyses were adjusted for median income, unemployment rate, and percent of population in urban areas.

**Results:**

In adjusted analyses, household firearm prevalence is significantly associated with overall suicide rates (adjusted incidence rate ratio (IRRa) = 1.28, 95% confidence interval (CI): 1.18, 1.38) and firearm suicides rates (IRRa = 1.61, CI: 1.45, 1.80), but not with non-firearm suicide rates (IRRa = 1.05, 95% CI: 0.95, 1.16). By contrast, adjusted analyses find no relationship between suicide rates and antidepressant prescription rates. Findings from county-level analyses were consistent with state-level results.

**Conclusion:**

The prevalence of household firearm ownership is strongly and significantly associated with overall suicide rates, due to its association with firearm suicide rates. This association is robust to consideration of the role of antidepressant prescription rates. A relationship between antidepressant prescription rates and suicide rates was not observed before or after adjusting for firearm ownership.

## Background

On an average day in 2010, the last year for which national data are available, more than 100 Americans died by suicide; half used firearms (Centers for Disease Control and Prevention (CDC)). Suicide, the tenth leading cause of death among Americans in 2010 (Centers for Disease Control and Prevention (CDC)), occurs at substantially higher rates in rural compared with urban areas, largely due to higher rates of firearm suicide in rural areas (Singh and Siahpush 2002). Prior research using mortality and prescription data from the 1990s explains the rural-urban suicide gradient as largely due to the fact that (in the 1990s) newer antidepressants (largely SSRIs) were less likely to be prescribed in rural than in urban areas (Gibbons et al. 2005). Injury researchers, by contrast, have long observed that the rural-urban gradient in US suicide rates, both during the 1990s and presently, is largely explained by a pronounced gradient in firearm (but not non-firearm) suicide rates and, furthermore, that the firearm-suicide gradient is highly correlated with rates of household firearm ownership (Branas et al. 2004; Miller and Hemenway 1999).

These two key exposures – i.e., firearm ownership and antidepressant prescribing – have been adduced to explain geographic patterns of US suicide mortality more broadly as well. For example, as reviewed previously (Miller and Hemenway 1999;2012; Brent 2001) ecologic studies in the U.S. have linked variation in suicide rates with the distribution of household firearm ownership. These studies have evaluated variation across states, census divisions, and cities, as well as over time, and have found overall suicide rates and firearm suicide rates are positively associated with firearm ownership prevalence. Consistent with these ecologic observations, case-control studies have also linked exposure to firearms in the home with increased risk of suicide overall due to an increased risk of suicide by firearm (Brent 2001; Kellermann et al. 1992; Conwell et al. 2002; Cummings et al. 1997; Wiebe 2003; Shah et al. 2000; Dahlberg et al. 2004; Bailey et al. 1997; Grassel et al. 2003; Kung et al. 2003). Geographic (Gibbons et al. 2005; Grunebaum et al. 2004) and temporal (Olfson et al. 2003) variation in suicide rates has also been linked to variation in antidepressant prescription patterns. While some early studies reported an inverse association between antidepressant prescription rates and overall suicide rates (Grunebaum et al. 2004; Olfson et al. 2003), a later review of nineteen ecological studies from around the world found that less than half observed such an association (Baldessarini et al. 2007). At least one early US study that found an inverse association between antidepressant prescription rates and overall suicide (Olfson et al. 2003) noted that future studies should account for gun ownership if and when such measures became available.

The current study is, to our knowledge, the first to consider the joint contribution of rates of antidepressant prescribing *and* household gun ownership on suicide mortality in the United States.

## Methods

We assembled an extensive panel data set from the United States including rates of suicide, antidepressant prescriptions, firearm ownership, and other covariates of interest.

### Households with firearms

The proportion of adults living in households with at least one gun was obtained from the Behavioral Risk Factor Surveillance System (BRFSS) (Centers for Disease Control and Prevention (CDC) 2001), a large survey of U.S. adults conducted annually by the Centers for Disease Control and Prevention (CDC) since 1994. Designed primarily to produce state-level estimates, since 2002 BRFSS has also produced county level estimates for eligible counties. Data on gun ownership was collected from all 50 states in the years 2001, 2002, and 2004, and for 146 and 199 counties in 2002 and 2004, respectively. If an estimate for a county was available for only one year, that estimate was used. When estimates were available for both years, an averaged estimate was used. After combining the two years, 219 counties representing 49 states and the District of Columbia were available. These counties represent 7% of the 3140 counties in the United States, and 46% of the total population. Surveyors asked respondents (yes/no) if there “are any firearms kept in or around your home?” and instructed respondents to “please include weapons such as pistols, shotguns, and rifles; but not BB guns, starter pistols, or guns that cannot fire. Include those kept in a garage, outdoor storage area, or motor vehicle”. The BRFSS has not included questions on gun ownership since 2004.

### Suicides

Annual counts of completed suicides for each state from 2001 through 2004, inclusive, were ascertained from the Center for Diseases Control’s National Vital Statistics System (Centers for Disease Control and Prevention (CDC)). Annual suicide counts were also collected separately by method: firearm and non-firearm. For secondary analyses, suicide counts were collected for counties from 2001 through 2005. Suicides from 2005 were included to improve the power of county level analyses. Due to suppression of small cell counts, deaths were summed over the five years. Counts were collected for total suicides and separately by method: firearm and non-firearm.

### Antidepressants

Antidepressant prescription data came from the IMS Health National Prescription Audit Database. Prescription data constitute a nationally representative random sample of pharmacies (stratified by type, size and region) and capture approximately 70% of all prescriptions filled in the US. Rates were calculated as number of prescriptions per 100,000 people.

### Other covariates

Additional covariates identified as potential confounders in prior work included in this study are median income (Hawton et al. 2001), unemployment rate (Lundin and Hemmingsson 2009), and the percent of the population living in an urban area (Singh and Siahpush 2002). These covariates were treated as continuous variables. Median income and unemployment rates are available annually from the Bureau of Labor Statistics. Annual estimates of the percent of population living in urban areas were calculated by assuming that the rate of change between 1990 and 2000 (available census estimate) was constant. For county-level analyses, Rural Urban Continuum Codes (RUCC) were used as an ordinal scale to measure the percent of the population living in urban areas. These codes are assigned by the US Department of Agriculture (US Department of Agriculture (USDA)).

#### Analysis

Mixed-effects regression models estimated incidence rate ratios and corresponding 95% confidence intervals. A negative-binomial distribution was selected to allow for over-dispersion of suicide rates about the mean. The regression models included a randomly varying intercept to allow for natural heterogeneity in suicide rates across states and to account for the correlation among the suicide rates over the five measurement occasions (2001-2004 in state-level analyses). The effect of antidepressant prescription rates (prescriptions per 100,000 people), and the effect of household firearm ownership rates (percent of households with firearms) were considered fixed effects; confounders were also treated as fixed effects. All models included dummy variables for each year in the study period to account for possible temporal trends. To distinguish cross-state and within-state variation, separate effects (of prescription rates and household firearm ownership) were estimated by including two versions of each factor in the models: (1) the mean of the factor over the four years and (2) deviations from the factor mean at each year. Specifically, the general form of the mixed models for the suicide rates was as follows:

$$\begin{array}{l} \mathrm{Log}\left(\mu_{\mathrm{ij}}\right)=\beta_{0}+b_{1}+\beta_{1}+\mathrm{Avg}F_{i}+\beta_{2}+\left(F_{\mathrm{ij}}‒\mathrm{Avg}F_{i}\right)\\ \;+\beta_{3}+\mathrm{Avg}A_{i}+\beta_{4}+\left(A_{i}‒\mathrm{Avg}A_{i}\right), \end{array}$$

where μ_ij_ denotes the suicide rate in the ith state at the jth occasion, F_ij_ (A_ij_) denotes the firearm ownership rate (antidepressant prescription rate) in the ith state at the jth occasion, and AvgF_i_ (AvgA_i_) is the mean firearm ownership rate (mean antidepressant prescription rate) over the four occasions. In this model, b_i_ is the random effect, allowing for heterogeneity in the suicide rates across states; β_1_ is the cross-state estimate of the effect of firearm ownership whereas β_2_ is the within-state estimate of the effect of firearm ownership. Similarly, β_3_ is the cross-state estimate of the effect of antidepressant prescriptions whereas β_4_ is the within-state estimate of the effect of antidepressant prescriptions. When exponentiated, the β’s can be interpreted as incidence rate ratios. Finally, we note that the fitted models also included the effects of confounders and indicator variables for time. Models were fit using PROC GLIMMIX in SAS.

For ease of interpretation of the regression coefficients, antidepressant prescription rates, household firearm ownership, and other covariates were standardized to a mean of zero and a standard deviation of one. Thus the reported incidence rate ratios correspond to a one standard deviation departure from the mean of the factor.

Secondary cross-sectional analyses were run on the available county level data aggregated over time. As with state-level analyses, negative-binomial regression models were repeated in the same pattern as primary analyses; models were fit using PROC GENMOD in SAS.

Results presented pertain to cross-state variation as suicide rates, firearm ownership, and prescription rates all changed trivially from one year to the next within any one state and no factor was significantly related to suicide rates in analyses that focused on within-state variation (not shown). Secondary cross-sectional analyses were run on the available county level data aggregated over time. As with state-level analyses, negative-binomial regression models were repeated in the same pattern as primary analyses; models were fit using PROC GENMOD in SAS.

For ease of interpretation of the regression coefficients, antidepressant prescription rates, household firearm ownership, and other covariates were standardized to a mean of zero and a standard deviation of one. Thus the reported incidence rate ratios correspond to a one standard deviation departure from the mean of the factor.

## Results

Over the study period, 2001-2004, there were 126,200 suicides in the US of which 67,634 involved firearms (53%). Firearm suicide rates varied 8.4 fold across the 50 states, whereas non-firearm suicide rates varied 2.7 fold (Table 1). The percent of people living in homes with firearms ranged widely, from 8% to 63%. Annual antidepressant prescription rates varied from 20,444 prescriptions per 100,000 people to 68,676 per 100,000 people.

### State-level analyses

In unadjusted analyses, suicide rates overall (IRR = 1.20, 95% CI: 1.14, 1.27) and by firearms (IRR = 1.51, 95% CI: 1.40, 1.63), were higher where a greater proportion of adults lived in households containing a gun (Table 2). These associations remained robust to adjustment for antidepressant prescription rates, income, unemployment, and urbanization (IRRa for suicide overall = 1.28, 95% CI: 1.18, 1.38; IRRa for suicide by firearm = 1.61, 95% CI: 1.45, 1.80). By contrast, non-firearm suicide rates were not significantly associated with household firearm ownership in either adjusted or unadjusted analyses.

**Table 1:** **State and county characteristics aggregated over the 5 year time period (2001-2005)**

	States (n = 50)	Counties (n = 220)
Percent of the population	100%	46%
	Mean (SD) [Range]	Mean (SD) [Range]
Suicides per 100,000 people	10.9 (3.4) [6.1 – 19.9]	11.2 (3.6) [4.3 – 27.2]
Firearm suicides per 100,000 people	7.0 (2.9) [1.7 – 14.7]	5.7 (2.9) [0.9 – 21.3]
Non-firearm suicides per 100,000 people	5.4 (1.3) [2.8 – 9.5]	5.5 (1.6) [2.1 – 10.0]
Percent of households with firearms	38.9 (13.9) [8.0 – 63.1]	29.5 (13.7) [1.7 – 65.1]
Annual antidepressant prescription fills per 100,000 people	48,426.2 (10,270.8) [20,444.1 – 68,675.6]	57,606.3 (22,257.7) [4,498.6 – 126,778.0]
Percent unemployment	5.1 (1.1) [2.8 – 6.4]	5.2 (1.4) [2.6-16.3]
Median income (USD)	51,595.4 (7,792.4) [36,543 – 70,645]	46,815.0 (10,217.0) 005B27,608.6 – 81,112.8]

**Table 2:** **Cross-state associations between suicide rates (total and method-specific)** **and rates of household firearm ownership** **and antidepressants prescription** **, 2001-2004**

	All suicide			Firearm suicide			Non-firearm suicide		
	IRR*	95% CI	P-value	IRR*	95% CI	P-value	IRR*	95% CI	P-value
Crude analyses									
Household firearm ownership	1.20	(1.14, 1.27)	<.0001	1.51	(1.40, 1.63)	<.0001	0.97	(0.91, 1.03)	0.3454
Antidepressant prescription rate	1.00	(0.93, 1.08)	0.9965	1.04	(0.90, 1.20)	0.5570	0.96	(0.90, 1.03)	0.2602
Multivariate analyses									
Household firearm ownership	1.28	(1.18, 1.38)	<.0001	1.61	(1.45, 1.80)	<.0001	1.05	(0.95, 1.16)	0.3703
Antidepressant prescription rate	0.96	(0.90, 1.02)	0.1858	0.93	(0.85, 1.02)	0.1437	1.00	(0.92, 1.08)	0.9855
% Population living in urban area	1.07	(0.98, 1.18)	0.1329	1.10	(0.97, 1.25)	0.1414	1.08	(0.96, 1.21)	0.1909
Median income	0.99	(0.95, 1.02)	0.4958	0.98	(0.93, 1.02)	0.2988	1.02	(0.96, 1.07)	0.5483
Unemployment rate	1.00	(0.98, 1.03)	0.9324	0.99	(0.96, 1.02)	0.5931	1.00	(0.97, 1.04)	0.8628

*IRR’s for Household Firearm Ownership (Antidepressant Prescription Rate) and Antidepressant Prescriptions are the relative increase in suicides for one standard deviation increase away from the mean rate.

*Abbreviations:* IRR, Incidence Rate Ratio; CI, Confidence Intervals.

In adjusted (as well as unadjusted) analyses, antidepressant prescription rates were not associated with overall suicide rates (IRRa = 0.96, CI: 0.90, 1.02) (Table 2), with firearm suicide rates (IRRa = 0.93, CI: 0.85, 1.02), or with non-firearm suicide rates (IRRa = 1.00, CI: 0.92, 1.08).

### County-level analyses

Findings at the county level (Table 3) were similar to state-level observations. Adjusted analyses indicated higher household firearm ownership was associated with higher overall suicides (IRRa = 1.23, 95% CI: 1.18, 1.28) and firearm suicides (IRRa = 1.60, 95% CI: 1.51, 1.69), but not non-firearm suicides. Antidepressant prescription rates were not associated with total suicide rates or method-specific suicide rates.

**Table 3:** **Cross-county associations between suicide rates (total and method-specific) and rates of household firearm ownership and antidepressants prescription, 2001-2005**

	All suicide			Firearm suicide			Non-firearm suicide		
	IRR*	95% CI	P-Value	IRR*	95% CI	P-Value	IRR*	95% CI	P-Value
Multivariate analyses									
Household firearm ownership	1.23	(1.18, 1.28)	<.0001	1.60	(1.51, 1.69)	<.0001	1.00	(0.95, 1.04)	0.8409
Antidepressant prescription rate	1.01	(0.97, 1.04)	0.6745	1.02	(0.97, 1.07)	0.5214	1.00	(0.96, 1.04)	0.9767
Rural urban continuum code	0.98	(0.94, 1.02)	0.3563	0.92	(0.87, 0.97)	0.0016	1.02	(0.97, 1.07)	0.3789
Median income	0.93	(0.89, 0.96)	0.0001	0.88	(0.83, 0.93)	<.0001	0.97	(0.92, 1.01)	0.1738
Unemployment rate	0.97	(0.93, 1.00)	0.0681	0.97	(0.92, 1.03)	0.3064	0.95	(0.90, 0.99)	0.0281

*IRR’s for Household Firearm Ownership (Antidepressant Prescription Rate) and Antidepressant Prescriptions are the relative increase in suicides for one standard deviation increase away from the mean rate.

*Abbreviations:* IRR, Incidence Rate Ratio; CI, Confidence Intervals.

## Discussion

Our study is the first to simultaneously evaluate the effects of firearm ownership and antidepressant prescription rates on the distribution of suicide rates across the United States. Consistent with prior ecologic and individual level studies from the United States (Miller and Hemenway 1999; Miller et al. 2012; Brent 2001; Kellermann et al. 1992; Conwell et al. 2002; Cummings et al. 1997; Wiebe 2003; Shah et al. 2000; Dahlberg et al. 2004; Bailey et al. 1997; Grassel et al. 2003; Kung et al. 2003), we find a robust association between firearm ownership and suicide. Moreover, this association remains significant and undiminished in magnitude after adjusting for antidepressant prescription rates, median income, unemployment rates, and urbanization. This covariation with overall suicides appears to be driven by covariation with firearm suicides.

By contrast, we do not observe a significant relationship between antidepressant prescription rates and completed suicide. Our study finds that where more antidepressants are prescribed per capita, suicide rates are neither higher nor lower than in states with lower prescribing rates, consistent with Gunebaum et al’s (2004) adjusted analyses, but contrary to some findings from Olfson et al. (2003) Olfson et al. observed that regions with average higher adolescent use of antidepressants in 2000, compared with use in 1990, had lower suicide rates in 2000. In their discussion, Olfson et al. noted that because other factors related to suicide risk also changed over the period, studies, such as ours, that examine whether the association between antidepressants and suicide that they report might be confounded by gun prevalence were needed.

Like Gibbons et al, (2005) we did not find a relationship between rates of total antidepressants prescribed and suicide. Gibbons, however, using data from the 1990s, found an inverse cross-sectional association between suicide and rates of prescriptions for new antidepressants, mostly selective serotonin reuptake inhibitors (SSRIs), when adjusting for tricyclic antidepressants (TCAs) prescription rates (presented by Gibbons as the ratio of newer to older antidepressant prescription rates). Repeating Gibbons’ analyses with our more recent data also finds that the ratio is associated with overall suicide rates. However, when we adjust for firearm ownership, which, like the ratio of newer to older antidepressants, is highly correlated with the rural-urban gradient, the association between the ratio and overall suicide rates, as well as firearm suicide rates, is rendered null (see Tables 4 and 5) -- suggesting that prior reports of an inverse relation between newer antidepressant prescription rates and suicide were confounded by the failure to control for firearm ownership.

**Table 4:** **Cross-state associations between suicide rates (total and method-specific) and household firearm ownership and antidepressants prescription rates, 2001-2005**

	All suicide			Firearm suicide			Nonfirearm suicide		
	IRR*	95% CI	P-value	IRR*	95% CI	P-value	IRR*	95% CI	P-value
Crude analyses									
NonTCA^:TCA ratio	0.85	(0.76, 0.95)	0.0040	0.69	(0.57, 0.84)	0.0002	1.07	(0.96, 1.18)	0.2270
Multivariate analyses									
Household firearm ownership	1.29	(1.18, 1.40)	<.0001	1.67	(1.49, 1.88)	<.0001	1.04	(0.94, 1.16)	0.4441
NonTCA^:TCA ratio	1.02	(0.91, 1.14)	0.7462	1.05	(0.90, 1.22)	0.5392	1.02	(0.89, 1.17)	0.8198
% Population living in urban area	1.09	(1.01, 1.19)	0.0293	1.14	(1.02, 1.27)	0.0211	1.08	(0.98, 1.20)	0.1407
Median income	0.99	(0.95, 1.02)	0.4770	0.97	(0.93, 1.02)	0.2758	1.01	(0.96, 1.07)	0.6421
Unemployment rate	1.01	(0.98, 1.03)	0.6227	0.99	(0.96, 1.03)	0.6932	1.01	(0.98, 1.05)	0.5231

*IRR for Household Firearm Ownership and Antidepressant Prescriptions are the relative increase in suicides for one standard deviation away from the mean rate.

^Non-TCA are primarily SSRIs (SNRIs constitute the second most commonly prescribed class).

*Abbreviations:* IRR, Incidence Rate Ratio; CI, Confidence Intervals; TCA, Tricyclic Antidepressants.

**Table 5:** **Cross-county associations between suicide rates (total and method-specific) and household firearm ownership and antidepressants prescription rates, 2001-2005**

	All suicide			Firearm suicide			Nonfirearm suicide		
	IRR*	95% CI	P-value	IRR*	95% CI	P-value	IRR*	95% CI	P-value
Crude analyses									
NonTCA^:TCA ratio	0.90	(0.86, 0.93)	<.0001	0.80	(0.75, 0.86)	<.0001	1.00	(0.96, 1.04)	0.9867
Multivariate analyses									
Household firearm ownership	1.23	(1.18, 1.28)	<.0001	1.60	(1.52, 1.70)	<.0001	1.00	(0.95, 1.05)	0.9658
NonTCA^:TCA ratio	1.01	(0.96, 1.06)	0.6820	1.01	(0.95, 1.08)	0.7400	1.03	(0.97, 1.09)	0.3802
% Population living in urban area	0.98	(0.94, 1.02)	0.3824	0.92	(0.87, 0.97)	0.0019	1.03	(0.98, 1.08)	0.3042
Median income	0.92	(0.87, 0.96)	0.0005	0.87	(0.81, 0.93)	<.0001	0.95	(0.90, 1.01)	0.0997
Unemployment rate	0.96	(0.93, 1.00)	0.0453	0.97	(0.92, 1.02)	0.2156	0.95	(0.91, 0.99)	0.0279

*IRR for Household Firearm Ownership and Antidepressant Prescriptions are the relative increase in suicides for one standard deviation away.

^Non-TCA are primarily SSRIs (SNRIs constitute the second most commonly prescribed class).

*Abbreviations:* IRR, Incidence Rate Ratio; CI, Confidence Intervals; TCA, Tricyclic Antidepressants from the mean rate.

Our findings should be considered in light of several possible limitations. First, as an ecological study, our study cannot identify whether the people who completed suicide tended, in fact, to live in homes with guns or to take antidepressant medication. Second, although our state and county-level analyses produce congruent results, our county-level findings could be influenced by county selection. The counties in our study were selected for a single reason: firearm estimates were available in the BRFSS. There is no *a priori* reason, however, to think this would introduce bias. Indeed, our counties, which tended to be more urban and to have lower household ownership rates than the US as a whole, also had slightly lower suicide rates, driven by fewer firearm suicides per capita (a pattern consistent with our general finding that where there are more guns there are more suicides).

## Conclusions

The current study does not, it should be underscored, rule out the possibility that antidepressants may have a beneficial (or a harmful) effect on suicide rates, but rather indicates that prior ecological evidence of the effectiveness of antidepressants in reducing US suicide rates were confounded by a failure to adjust for firearm ownership rates. The current paper also demonstrates the robust relationship between firearms and suicide, adds to the growing literature connecting access to firearms in the home with completed suicides, and lends further support to the importance of integrating means restriction approaches into US suicide prevention policy.
